# Surgical Management of Cardiac Masses in Right Atrium Among Bone Sarcoma Pediatric Patients With Totally Implanted Ports

**DOI:** 10.3389/fonc.2022.926387

**Published:** 2022-06-16

**Authors:** Chenliang Zhou, Yiyun Wang, Zonghui Chen, Guowei Qian, Wenxi Yu, Yong Wang, Shuier Zheng, Zan Shen, Hongtao Li, Yonggang Wang

**Affiliations:** ^1^ Department of Oncology, Shanghai Jiao Tong University Affiliated Sixth People’s Hospital, Shanghai, China; ^2^ Department of Emergency, Shanghai United Family Hospital, Shanghai, China; ^3^ Department of Cardiovascular Surgery, Shanghai Jiao Tong University Affiliated Sixth People’s Hospital, Shanghai, China; ^4^ Department of Ultrasound in Medicine, Shanghai Jiao Tong University Affiliated Sixth People’s Hospital, Shanghai, China

**Keywords:** sarcoma, catheter-related right atrial thrombosis, surgery, cardiac metastasis, totally implanted ports

## Abstract

**Introduction:**

Totally implanted ports (PORTs) have been widely used among patients with malignancy. Cardiac metastasis secondary to bone sarcoma and catheter-related right atrial thrombosis (CRAT) can be both present as cardiac masses. However, these two cardiac masses share very similar imaging characteristics.

**Methods:**

The features, treatments, and outcomes of 5 bone sarcoma pediatric patients with PORTs who suffered from cardiac masses in the right atrium were analyzed. Clinical data and histological characteristics of cardiac masses were also recorded.

**Results:**

Among 928 patients with malignancy and PORTs, 5 bone sarcoma pediatric patients were found to have cardiac masses in the right atrium. The catheter tips were located in the right atrium of 4 patients and the superior vena cava-right atrium junction (CAJ) of 1 patient. Four patients with good response to anti-tumor treatment had received surgical lumpectomies for pathologic identification and mass excision, with cardiac metastases among 1 patient and thromboses among 3 patients. The median time from venous access port implantation to cardiac mass detection for CRAT was 6.3 months (range: 4.7–6.8 months) and to diagnosis of or possible cardiac metastasis was 13.3 months (range: 11.2–15.4 months).

**Conclusion:**

The placement of a catheter tip into the right atrium should be avoided. The time from PORTs implantation to cardiac mass detection might serve as a potential tool to differentiate cardiac metastasis from CRAT. Surgical management may be an effective treatment for bone sarcoma pediatric patients who had good response to anti-tumor treatment and suffered from cardiac masses in the right atrium.

## Introduction

Bone sarcomas are rare neoplasms, accounting for less than 0.2% of all cancers (1), spread hematogenously, with the lung and bone being the most common metastatic sites (2). Metastases from bone sarcoma to the heart are rare (3).

Totally implanted ports (PORTs) have been widely used among bone sarcoma patients. Catheter-related right atrial thrombosis (CRAT) is one of the most serious complications with an estimated incidence of 5.4%–12.5% (4, 5). Nevertheless, studies on CRAT secondary to PORTs among bone sarcoma patients are lacking.

Cardiac metastasis secondary to bone sarcoma and CRAT can be both presented as cardiac masses (6), with similar characteristics of calcification or ossification in CT and hyperechogenicity on echocardiogram. However, they should be differentiated when patients suffer from cardiac masses, as they require different treatments (7–10).

To the best of the authors’ knowledge, we firstly described a series of bone sarcoma patients with PORTs who had cardiac masses in the right atrium. Moreover, we also assessed their treatments and outcomes.

## Methods

Patients with PORTs admitted to the department of oncology in Shanghai Jiao Tong University Affiliated Sixth People’s Hospital from January 2018 to December 2020 were identified (n=928). Among these patients, 5 patients with cardiac masses in the right atrium were included. Patients’ demographics, surgical, and histological data were collected with median follow-up of 16.2 months (range, 3.0–24.5 months) from cardiac mass detection.

An implantable venous access port, consisting of a chamber connected to a catheter, was placed under the skin. Initial venous access was established by using the percutaneous approach with ultrasound. The catheter was threaded into the axillary vein or the jugular vein. Then, the subcutaneous reservoir was placed in a pocket created anterior to the pectoralis major muscle in the sub-clavicular region and accessed *via* a specific needle through the intact skin. The catheter tip position was verified radiologically with intraoperative fluoroscopy.

The surgical management of cardiac masses was performed by an experienced cardiac surgery team. Surgeons dissociated the right femoral artery and vein, offered systemic anticoagulation with heparin, and established cardiopulmonary bypass with the femoral artery for supply and the femoral vein for drainage. Surgeries were performed in the 30-degree left lateral decubitus position and with double-lumen tube ventilation after a 4-cm long anterolateral thoracotomy in the 4th intercostal space on the right side. Meanwhile, a 1-cm incision was conducted on the mid-axillary line of the 4th intercostal space for thoracoscopic placement and a 1-cm incision on the mid-axillary line of the third intercostal space to drain the superior vena cava. Opening right atrium revealed that the masses were mainly attached to the right atrium wall. Surgeons cut along the edge of the tumor and part of the right atrium wall for definitive diagnosis with minimal damage and maximal mass removal.

Pulmonary CT with contrast, trans-thoracic echocardiogram (TTE), and PET-CT or bone scans were conducted for all patients. The changes in cardiac masses were observed dynamically, as shown in pulmonary CT and TTE. Transesophageal echocardiography (TEE) was also carried out for patients who received surgery for cardiac masses.

This study was approved by the Institutional Review Board of Shanghai Jiao Tong University Affiliated Sixth People’s Hospital, and informed consent was obtained from all patients (Approval no. 2021–080). This study included a small number of patients and thus, was not deemed suitable for further statistical analyses.

## Results

### Patient Demographics

In total, 928 patients with tumors received PORTs from January 2018 to December 2020 in our hospital. The pathologic results revealed 425 patients suffered from malignant tumors of epithelial origin, 302 from bone sarcomas, and 201 from soft tissue sarcomas. Moreover, patients (3 females and 2 males; children 8 – 15 years old when diagnosed) with cardiac masses in the right atrium were diagnosed with bone sarcoma. The clinic characteristics and treatments are described in **Table 1**. The primary tumors were all located in the extremities. At presentation, 4 osteosarcoma patients were diagnosed at Enneking stage IIB, while the remaining patient with undifferentiated small round cell sarcoma was diagnosed at Enneking stage IIA.

**Table 1:** Clinical and histological features of primary tumors in bone sarcoma pediatric patients.

Patient No./Sex	Age at diagnosis	Comorbidities	Histologic subtype of primary tumor	Primary tumor site	Size of primary tumor (cm)	Enneking stage	Regimen of neoadjuvant chemotherapy	Necrosis of Huvos grade after neoadjuvant chemotherapy
1/F	8	None	Undifferentiated small round cell sarcoma	Femur	6×3.9×2	IIA	VAC/IE	III
2/F	13	None	Osteosarcoma	Tibia	15.8×7.8×6.1	IIB	MAPI	III
3/M	13	None	Osteosarcoma	Tibia	3.6×3.2×3	IIB	MAPI	I
4/F	8	None	Osteosarcoma	Humerus	12.2×7.3×7.5	IIB	MAPI	III
5/M	15	None	Osteosarcoma	Humerus	8.4×8.9×29.1	IIB	MAPI	II

F, female; M, male; VAC, vincristine+ doxorubicin+ cyclophosphamide; IE, ifosfamide+ etoposide; MAPI, methotrexate+ cisplatin+ doxorubicin+ ifosfamide.

The first-line treatment was prescribed according to the NCCN guideline. Generally, MAPI was administered for osteosarcoma and VAC/IE for undifferentiated small round cell sarcoma. Three patients had good response to neoadjuvant chemotherapy with tumor necrosis of Huvos grade III.

#### Features of Cardiac Masses in Bone Sarcoma Patients

Features of cardiac masses in patients with bone sarcoma are shown in **Table 2**. Five patients had received chemotherapeutics *via* PORTs. The catheter tips were located in the right atrium of 4 patients and the superior vena cava-right atrium junction (CAJ) of 1 patient. Only the patient who received palliative treatment had shortness of breath and dyspnea after activities, 3 patients received adjuvant chemotherapy, and 1 patient was asymptomatic. Cardiac masses could be found by CT as calcification or ossification in 3 patients and hyperechogenicity on an echocardiogram in all patients. The radiological data and operative field were presented in the **Supplement Figures**.

**Table 2:** Features of cardiac masses in pediatric patients with bone sarcoma.

Patient No./Sex	Tips of PORTs	Time from implantation of PORTs to cardiac mass detection (month)	Phase of primary tumor treatment	Symptom	Calcification or ossification *via* computed tomography	Size of cardiac mass (cm)	Echo by echocardiogram	D-dimer before treatment (mg/L FEU)	ALP before treatment (U/L)
1/F	RA	4.7	Adjuvant chemotherapy	Asymptomatic	No	2.0×0.9	Hyperechogenic	2.16	156
2/F	RA	11.2	Follow-up period	Asymptomatic	Yes	1.2×0.9	Hyperechogenic	0.49	110
3/M	RA	6.3	Adjuvant chemotherapy	Asymptomatic	Yes	2.8×1.2	Hyperechogenic	1.89	140
4/F	RA	6.8	Adjuvant chemotherapy	Asymptomatic	Yes	2.0×1.7	Hyperechogenic	0.26	257
5/M	CAJ	15.4	Palliative treatment period	Shortness of breath and dyspnea after activities	No	2.5×2.5	Hyperechogenic	0.70	110

CVC, central venous catheters; RA, right atrium; CAJ, superior vena cava-right atrium junction.

#### Treatments and Outcomes of Cardiac Masses in Bone Sarcoma Patients

Treatments and outcomes of cardiac masses in patients with bone sarcoma are shown in **Table 3**. Anticoagulation for 4 patients (2 receiving adjuvant chemotherapy, 1 palliative treatment, and 1 during follow-up) and surgical removal for 1 patient (during adjuvant chemotherapy) were prescribed as primary treatment. The mass increased in patients with palliative treatment (1/4). Besides, the size of cardiac masses did not change after anticoagulation in patients during adjuvant chemotherapy or the follow-up period (3/4). Therefore, they received surgical removal as rescue treatment. Among all 4 patients with surgical removal (1 as primary treatment and 3 as rescue treatment), their cardiac masses were not adherent to the catheter tips and there was no surgical complication. The pathology revealed that the cardiac masses were cardiac metastasis in 1 patient in the follow-up period but thrombus in 3 patients in the adjuvant chemotherapy period. The cardiac metastasis patient survived from cardiac mass detection (24.5 months) with effective anti-tumor treatment and without cardiac metastasis recurrence. The patients diagnosed with thrombus had no recurrence of cardiac thrombus during follow-up. The patient receiving palliative treatment had pulmonary, bone, and soft tissue metastasis. He had already been refractory to anti-tumor therapy and anticoagulation of cardiac masses. Therefore, his cardiac mass may be more likely cardiac metastasis. He died of extensive metastasis 3 months after cardiac mass detection.

**Table 3:** Treatments and outcomes of cardiac masses in pediatric patients with bone sarcoma.

Patient No./Sex	Anticoagulation for cardiac mass	Time of anticoagulation (month)	Response to anticoagulation	Surgery for cardiac mass	Catheter tips adherent to cardiac mass	Pathology of cardiac mass	Extra therapy for metastatic malignancy	Extracardiac metastasis at cardiac mass detection	Follow-up after cardiac mass detection (month)	Survival
1/F	Yes	3	Unchanged	Yes	No	Thrombus	–	No	22.3	Yes
2/F	Yes	1	Unchanged	Yes	No	Metastasis of osteosarcoma	GT/TKI	No	24.5	Yes
3/M	Yes	0.5	Unchanged	Yes	No	Thrombus	–	No	16.2	Yes
4/F	No	–	–	Yes	No	Thrombus	–	No	15.3	Yes
5/M	Yes	3	Increased	No	–	–	TKI	Pulmonary, bone, brain, and soft tissues	3.0	No

GT, gemcitabine+ docetaxel; TKI, Tyrosine Kinase Inhibitor.

The median time from PORTs implantation to cardiac mass detection for CRAT was 6.3 months (range: 4.7–6.8 months) and to diagnosis of or possible cardiac metastasis was 13.3 months (range: 11.2–15.4 months).

## Discussion

We investigated the features, treatments, and outcomes of cardiac masses among patients with PORTs. Cardiac masses in the right atrium were all observed in pediatric patients with bone sarcoma. The time from PORTs implantation to cardiac mass detection might serve as a potential tool to differentiate cardiac metastasis from CRAT. Surgical lumpectomy may be an effective way for diagnosis and management among this population.

Reports of cardiac masses among bone sarcoma patients are still rare (3, 11). In our center, all patients with cardiac masses found to be bone sarcoma were children. The rapid growth in pediatric patients, compared to adults, may partially explain the different incidences of cardiac masses between adults and children.

Several mechanisms may underline this complication, mainly direct endocardial injury by the tip of the catheter, although anti-tumor drugs, hypercoagulability, along with their actions on endothelial cells, may also be involved. Several authors have suggested that the risk of thrombosis increased when the catheter tip was located in the right atrium (7, 8). A prospective TEE study in patients with bone marrow transplantation showed that CRAT was found in 12.5% of the patients, all of whom were seen in a group with a CVC tip in the right atrium (5). Therefore, an assumption of placing the tip in the CAJ may be optimal for reducing the risk of CRAT. The catheter tips of 4 patients in our study were located in the right atrium. Given that children will grow older and taller, some surgeons would recommend positioning the tip of the catheter in the upper right atrium (12). Additionally, although the desired location of the catheter tip is at CAJ, the catheter tip may move with the movement and blood flow of the human body in the superior vena cava. Therefore, the ideal location is only a relative interval, and it is difficult to define an exact point (13). Moreover, the reason for the difference may also be explained by the technique between different surgeons. Inaccurate judgement of the position of the catheter tip, unreasonable design of the pathway, and the oversize subcutaneous reservoir may lead to the catheter tip reaching to right atrium.

Cardiac masses are clinical obstacles with significant heterogeneity in pathology and clinical presentation (6, 14). The patient with palliative treatment did not receive effective anti-tumor treatment and then suffered from the progression of metastasis. Moreover, his catheter tip was located in the CAJ and his cardiac masses were refractory to anticoagulation, therefore his cardiac masses are more likely cardiac metastasis. For other patients, to differentiate CRAT from cardiac metastasis is not always straightforward because both conditions could be presented as calcification or ossification in pulmonary CT and hyperechogenic on echocardiogram, especially among bone sarcoma patients with the definitive diagnosis made only by pathologic analysis.

One retrospective study revealed that the median time to thrombus detection was 77 days (range: 1–794 days) (7), which was similar to our result (6.3 months, range 4.7–6.8 months). Besides, CRAT seemed to require a shorter period of time to develop than cardiac metastasis (6.3 months vs 13.3 months) from our study, which indicates that the time from PORTs implantation to cardiac mass detection might serve as a potential tool to differentiate cardiac metastasis from CRAT. However, considering the limited number of patients from our study, future larger sample size studies are still required to further confirm our conclusion.

Despite their usefulness, the time to cardiac mass detection is not the gold standard to distinguish with certainty between CRAT and cardiac metastasis. Meanwhile, the evidence is not enough to establish the optimal treatment of CRAT and surgery is preferred in the majority of cases (15, 16). The patients receiving surgical lumpectomy in our study had good response to anti-tumor treatment without surgical complication.

While surgical management identified the pathology and excised masses, only limited conclusions were drawn from this study due to sample size limitation and the difference of the technique between different surgeons and thus further comprehensive studies, including clinical trials, should be considered. As cardiac masses in the right atrium of bone sarcoma patients is uncommon, relevant large clinical trials may be challenging.

The placement of a catheter tip into the right atrium should be avoided. The time from PORTs implantation to cardiac mass detection might serve as a potential tool to differentiate cardiac metastasis from CRAT. Surgical management may be an effective treatment for bone sarcoma pediatric patients who had good responses to anti-tumor treatment and suffered from cardiac masses in the right atrium.

## Data Availability Statement

The raw data supporting the conclusions of this article will be made available by the authors, without undue reservation.

## Ethics Statement

The studies involving human participants were reviewed and approved by Institutional Review Board of Shanghai Jiao Tong University Affiliated Sixth People’s Hospital. Written informed consent to participate in this study was provided by the participants’ legal guardian/next of kin.

## Author Contributions

All authors listed have made a substantial, direct, and intellectual contribution to the work, and approved it for publication.

## Conflict of Interest

The authors declare that the research was conducted in the absence of any commercial or financial relationships that could be construed as a potential conflict of interest.

## Publisher’s Note

All claims expressed in this article are solely those of the authors and do not necessarily represent those of their affiliated organizations, or those of the publisher, the editors and the reviewers. Any product that may be evaluated in this article, or claim that may be made by its manufacturer, is not guaranteed or endorsed by the publisher.

## Supplementary Material

Supplementary Figure S1 The radiographic data and operative field of patient 1. **(A)** On chest X-ray, catheter tip (blue arrow) was located in right atrium. **(B)** The levels of glucose metabolism of cardiac mass (red arrow) didn’t increase on positron emission tomography-computed tomography. The cardiac mass (red arrow) didn’t show calcification or ossification on computed tomography. **(C)** The cardiac mass showed hyperechogenicity on trans-thoracic echocardiogram (white plus sign). **(D)** The operative field showed the cardiac mass (yellow arrow).

Supplementary Figure S2 The radiographic data and operative field of patient 2. **(A)** On chest X-ray, catheter tip was located in right atrium (blue arrow). **(B)** The cardiac mass (red arrow) showed calcification or ossification on computed tomography. **(C)** The cardiac mass showed hyperechogenicity on trans-thoracic echocardiogram (white plus sign). **(D)** The operative field showed the cardiac mass (yellow arrow).

Supplementary Figure S3 The radiographic data and operative field of patient 3. **(A)** On chest X-ray, catheter tip was located in right atrium (blue arrow). **(B)** The levels of glucose metabolism of cardiac mass (red arrow) didn’t increase on positron emission tomography-computed tomography. The cardiac mass (red arrow) showed calcification or ossification on computed tomography. **(C)** The cardiac mass showed hyperechogenicity on trans- esophageal echocardiogram (white plus sign). **(D)** The operative field showed the cardiac mass (yellow arrow).

Supplementary Figure S4 The radiographic data and operative field of patient 4. **(A)** On chest X-ray, catheter tip was located in right atrium (blue arrow). **(B)** The cardiac mass (red arrow) showed calcification or ossification on computed tomography. **(C)** The cardiac mass showed hyperechogenicity on trans- esophageal echocardiogram (white plus sign). **(D)** The operative field showed the cardiac mass (yellow arrow).

Supplementary Figure S5 The radiographic data of patient 5. **(A)** On chest X-ray, catheter tip was located in superior vena cava-right atrium junction (blue arrow). **(B)** The cardiac mass (red arrow) didn’t show calcification or ossification on computed tomography. **(C)** The cardiac mass showed hyperechogenicity on trans-thoracic echocardiogram (white plus sign). **(D)** Pulmonary contrast-enhanced computed tomography showed the cardiac mass (yellow arrow) clearly.
