# Supplementary figures and images for: Surgical Management of Cardiac Masses in Right Atrium Among Bone Sarcoma Pediatric Patients With Totally Implanted Ports
Source: Front Oncol. 2022 Jun 16;12:926387. doi: 10.3389/fonc.2022.926387 (PMC9246414; doi:10.3389/fonc.2022.926387)

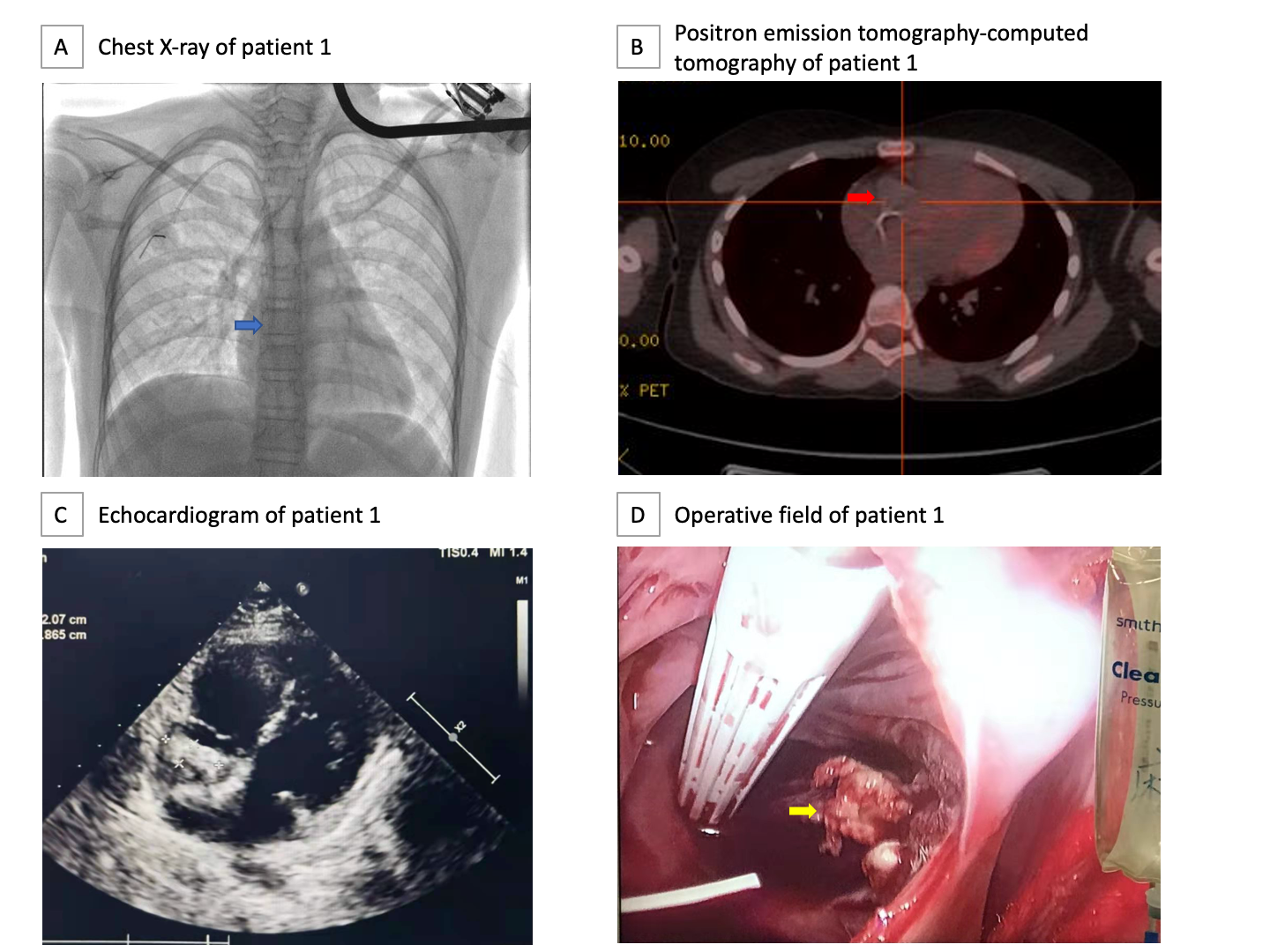

Supplement: Supplementary Figure S1 — The radiographic data and operative field of patient 1. (A) On chest X-ray, catheter tip (blue arrow) was located in right atrium. (B) The levels of glucose metabolism of cardiac mass (red arrow) didn’t increase on positron emission tomography-computed tomography. The cardiac mass (red arrow) didn’t show calcification or ossification on computed tomography. (C) The cardiac mass showed hyperechogenicity on trans-thoracic echocardiogram (white plus sign). (D) The operative field showed the cardiac mass (yellow arrow). [file Image_1.tiff]

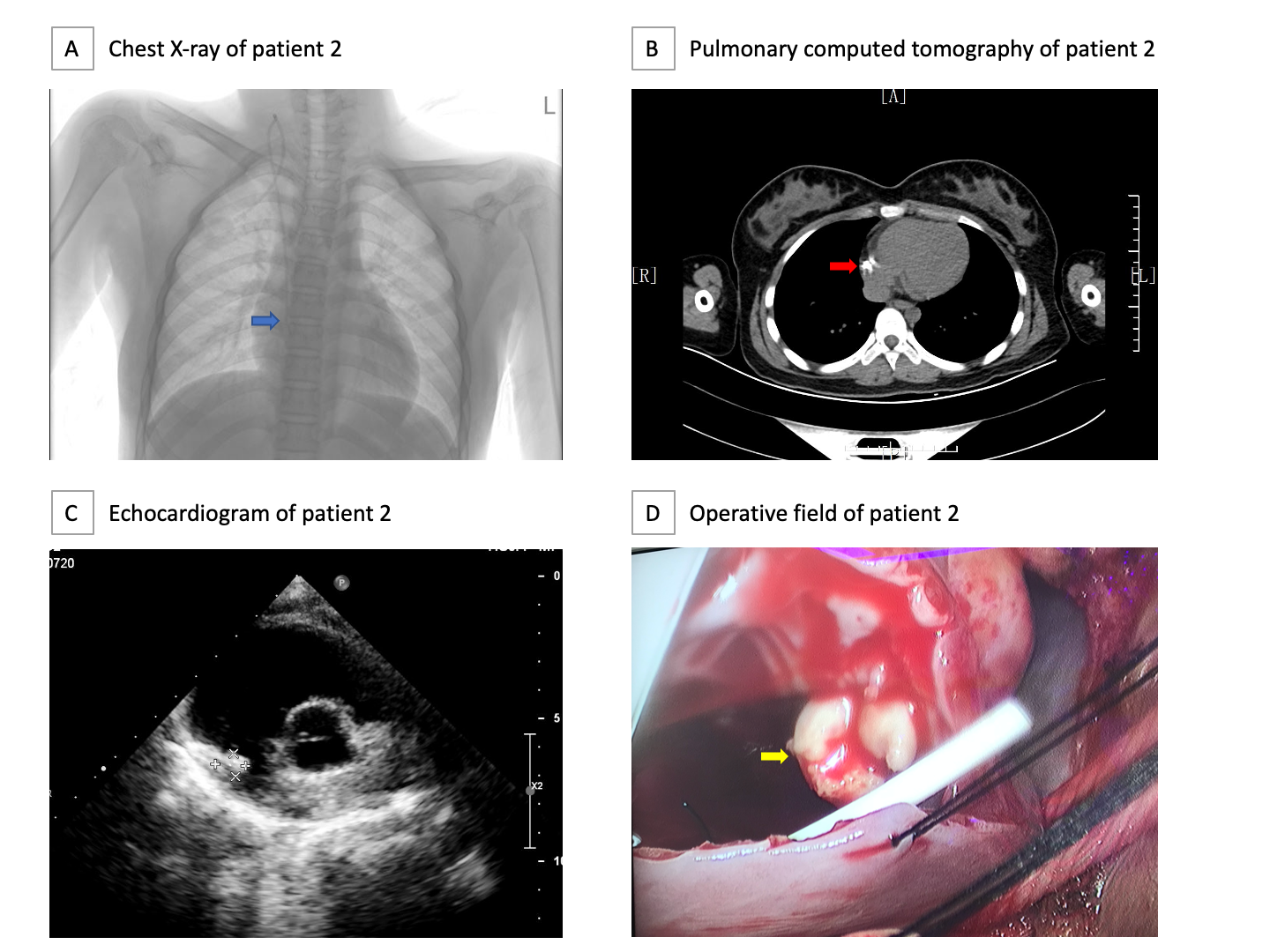

Supplement: Supplementary Figure S2 — The radiographic data and operative field of patient 2. (A) On chest X-ray, catheter tip was located in right atrium (blue arrow). (B) The cardiac mass (red arrow) showed calcification or ossification on computed tomography. (C) The cardiac mass showed hyperechogenicity on trans-thoracic echocardiogram (white plus sign). (D) The operative field showed the cardiac mass (yellow arrow). [file Image_2.tiff]

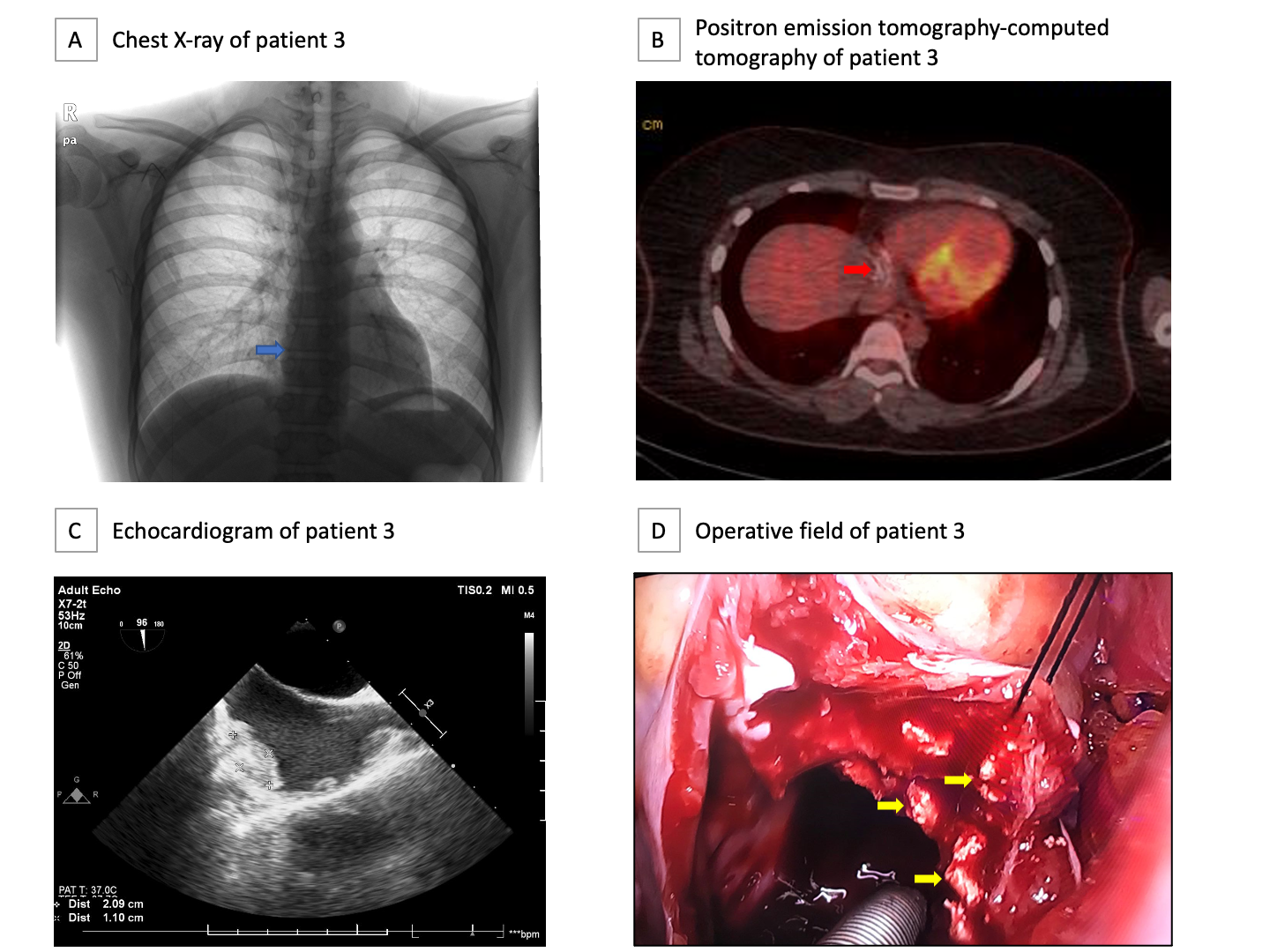

Supplement: Supplementary Figure S3 — The radiographic data and operative field of patient 3. (A) On chest X-ray, catheter tip was located in right atrium (blue arrow). (B) The levels of glucose metabolism of cardiac mass (red arrow) didn’t increase on positron emission tomography-computed tomography. The cardiac mass (red arrow) showed calcification or ossification on computed tomography. (C) The cardiac mass showed hyperechogenicity on trans- esophageal echocardiogram (white plus sign). (D) The operative field showed the cardiac mass (yellow arrow). [file Image_3.tiff]

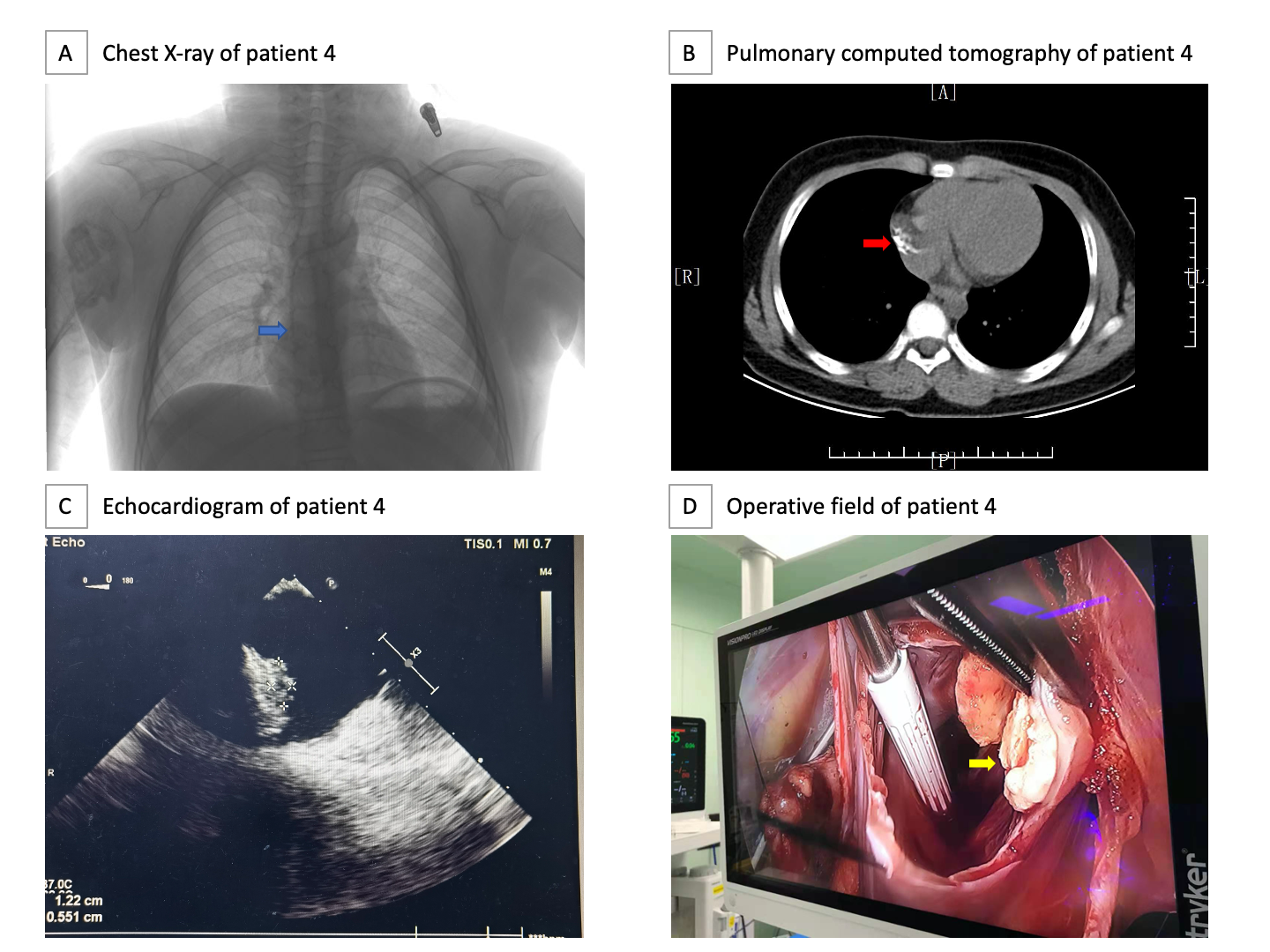

Supplement: Supplementary Figure S4 — The radiographic data and operative field of patient 4. (A) On chest X-ray, catheter tip was located in right atrium (blue arrow). (B) The cardiac mass (red arrow) showed calcification or ossification on computed tomography. (C) The cardiac mass showed hyperechogenicity on trans- esophageal echocardiogram (white plus sign). (D) The operative field showed the cardiac mass (yellow arrow). [file Image_4.tiff]

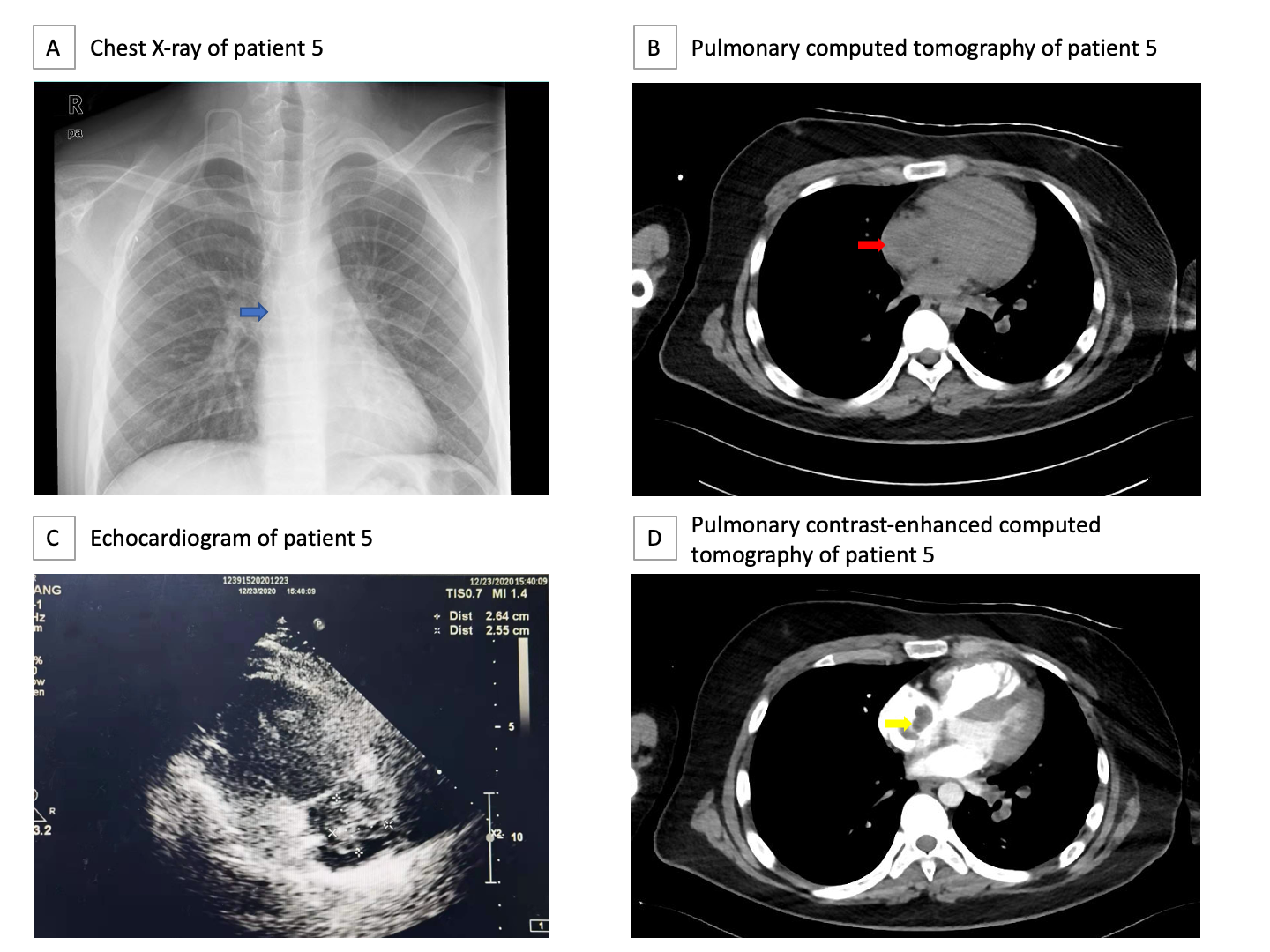

Supplement: Supplementary Figure S5 — The radiographic data of patient 5. (A) On chest X-ray, catheter tip was located in superior vena cava-right atrium junction (blue arrow). (B) The cardiac mass (red arrow) didn’t show calcification or ossification on computed tomography. (C) The cardiac mass showed hyperechogenicity on trans-thoracic echocardiogram (white plus sign). (D) Pulmonary contrast-enhanced computed tomography showed the cardiac mass (yellow arrow) clearly. [file Image_5.tiff]
